# The agricultural ethics of biofuels: climate ethics and mitigation arguments

**DOI:** 10.1007/s10202-012-0105-6

**Published:** 2012-03-28

**Authors:** Paul B. Thompson

**Affiliations:** Department of Philosophy, Michigan State University, East Lansing, MI 48824 USA

## Abstract

An environmental, climate mitigation rationale for research and development (R&D) on liquid transportation fuels derived from plants emerged among many scientists and engineers during the last decade. However, between 2006 and 2010, this climate ethic for pursuing biofuel became politically entangled and conceptually confused with rationales for encouraging greater use of plant-based ethanol that were both unconnected to climate ethics and potentially in conflict with the value-commitments providing a mitigation-oriented reason to promote and develop new and expanded sources of biofuel. I argue that the conceptual construct of technological trajectories provides a fecund approach to the ethical evaluation of R&D strategies in the case of plant-based liquid transportation fuels. The idea of a trajectory has a current use in the literature of science studies and aptly summarizes a number of themes that are critical to the evaluation of tools and techniques whose future shape, design, applications and potential consequences are necessarily somewhat speculative. In the case of biofuels, it is the imagined future trajectory that provides the basis for resistance to an emerging technology, rather than the present-day technical capabilities and the unexpected consequences of biofuel development.

## Abstract (de)

Die Erforschung und Entwicklung von Biokraftstoffen war im letzten Jahrzehnt maßgeblich durch Umwelt- und Klimaschutzziele motiviert. Gleichwohl gerieten diese Ziele in den letzten Jahren zunehmend in Konflikt mit anderen Initiativen, die die breite Nutzung von Äthanol aus Ackerpflanzen propagierten. Letztere scheinen bei genauer Betrachtung den Emissionsminderungszielen zum Klimaschutz auf konzeptioneller, ethischer und politischer Ebene zu widersprechen oder den Zielen zumindest nicht förderlich zu sein. Vor diesem Hintergrund wird ein Trajektorien-Modell zur technischen Entwicklung von Biokraftstoffen vorgeschlagen, das die ethische Evaluierung entsprechender Forschungsstrategien erlaubt. Das Prinzip der Pfadanalyse wird derzeit auch in der Fachliteratur diskutiert. Das Konzept sieht sich dabei vor der Herausforderung, eine Vielzahl kritischer Elemente und Prozesse zu evaluieren, deren zukünftige Entwicklung und Folgen notwendigerweise unsicher sind. Im Fall der Biokraftstoffe hängen Entscheidungen für entsprechende Entwicklungen mehr von ihren Zukunftsperspektiven als vom derzeitigen Stand der Technik und deren Nebenfolgen ab.

## Résumé

Pendant la décennie écoulée, l’exploration et la mise au point de biocarburants furent notamment dictées par les objectifs que l’on s’était proposés pour protéger l’environnement et lutter contre le réchauffement climatique. Toutefois, ces dernières années, ces objectifs se sont de plus en plus heurtés à d’autres initiatives propageant l’utilisation répandue d’éthanol extrait de fleurs des champs. Si on y regarde de plus près, ceux-ci semblent s’opposer, sur les niveaux conceptionnel, éthique et politique, aux objectifs de la réduction des émissions nocives dans la lutte contre le réchauffement climatique ou au moins ne pas être favorables à ceux-ci. Dans ce contexte, un modèle de trajectoires a été proposé pour la mise au point technique de biocarburants, permettant l’évaluation éthique de stratégies de recherche correspondantes. Le principe de l’analyse de chemin est actuellement aussi discuté dans la littérature spécialisée. Dans le cadre du projet, il faut donc procéder à l’évaluation d’une multitude d’éléments et de processus critiques dont la mise au point et les conséquences futures sont par définition aléatoires. Dans le cas des biocarburants, toute décision en faveur de telles mises au point dépend des perspectives d’avenir pronostiquées pour celles-ci plutôt que de l’état de la technique actuel et des effets accessoires qu’entraîne celui-ci.

## Introduction: technological trajectories

My early paper on the ethics of biofuel, entitled “A First Look,” used the idea of a “technological trajectory” to describe and analyze the key ethical issues associated with an emerging technology. The paper predicted that ethical analyses of biofuels focused on future impacts and unintended consequences would become embroiled in arcane methodological debates. I advised that despite the role of epistemic values and the philosophies of science in these debates, philosophers would be able to have little impact upon them and went on to argue that a focus on broad questions in the philosophy of agriculture would be a more fruitful approach (Thompson 2008). The preponderance of work on the ethics of biofuel since this paper has to some degree borne out my pessimism about methodological debates, but not my focus on the philosophy of agriculture. The paper significantly underplayed the significance of environmental impact in the ethical evaluation of biofuel and missed the ethical tension between food and fuel production entirely.

However, I will argue that my original account of technological trajectories nonetheless provides a fruitful approach to considering key ethical issues in biofuel development. Drawing on Don Ihde’s philosophy of technology, the original paper explained that a trajectory describes the expected development and maturation of a given cluster of tools and techniques, including and indeed emphasizing their interaction with and impact upon other technical practices, human affairs and the natural world. Trajectories recognize that the key elements in the configuration and impact of technical practices are unpredictable: the fate of a technological trajectory is at the mercy of the winds (not to mention intervening objects). But trajectories also reflect the fact that scientists, engineers, business executives and activists who are involved in R&D, technology promotion, regulation and also political resistance nonetheless envision the future that a given cluster of tools will or might bring about in at least a broadly sketched way (Ihde 1990).

The metaphor of a trajectory usefully integrates key themes in recent work on the ethics of emerging technology. In explicitly acknowledging the limitations inherent in projecting a given future for a given technology, the idea of a trajectory connects well with recent work on Collingridge’s dilemma and emerging technology (Collingridge 1980; van Merkerk and Smits 2008; Liebert and Schmidt 2010). The dilemma arises in connection with the fact that at the stages where one can have the most influence over the design and implementation of a technology, one cannot predict its effects. After the effects become known, many key technical parameters are “locked in.” Although a focus on trajectories does not eliminate the dilemma, it usefully indicates the way that some vague future is envisioned at each stage in the development of a technology. The trajectory may be altered by many things. It falls far short of a prediction. It is nonetheless an appropriate target for early-stage ethical analysis. In acknowledging the way that advocates or opponents of a technology work from a vaguely envisioned scenario for the future, the idea of a trajectory also connects well with recent work on the role of “imaginaries” in science and technology studies (Rabinow 1999; Mordini 2007). Indeed, because trajectories articulate the scenario that mobilizes scientists, engineers, investors and potential users into a network that will try to realize the development and implementation of a given technical means, they *are* “imaginaries” that script activity for individuals and groups integrated by the network to perform.

More pertinent to the ethics of technology, the rhetorical device of trajectories enables a normative analysis that provides a clear description of the ethical implications and commitments that are associated with pursuit and promotion of these imaginary and vague futures. At the same time, reference to a trajectory avoids both the attribution of intentions or agency to technical artifacts and the need to impute simplistic intentions to human beings whose commitments are almost always more equivocal and more complex. To talk of a trajectory is to utilize simplifying assumptions that enable discussion and debate over the future course of an emerging technology, but to do so without also imputing simple-mindedness to the human beings whose activity attempts to realize or “perform” the course of development and implementation that the notion of a trajectory suggests. A trajectory thus opens the space for critique of emerging technology well before its actual course of development and consequences can be known with confidence.

In this paper, the phrase *climate ethics* is used to refer broadly to arguments that identify and defend duties, responsibilities or costs and benefits in terms of mitigation of or adaptation to the environmental changes occurring in response to the rising atmospheric concentration of greenhouse gases. I begin by briefly summarizing the conceptual basis of an environmental ethics argument favoring biofuels as a mitigation strategy in response to increasing the levels of carbon in the global atmosphere. In March 2011, the Nuffield Council on Bioethics in Great Britain issued their report on the ethical evaluation of biofuels. The discussion below provides a brief review of the report and its key findings and then uses the framework of technological trajectories to characterize several distinct ways of imagining the technological future of biofuels. I argue that only some of the trajectories envisioned by the advocates of biofuel incorporate the elements of climate ethics to any significant degree. They might do this in technical terms by utilizing a life cycle analysis to select methods for producing or distributing biofuels that make relatively greater contribution to a reduction in greenhouse gas emissions. But, as will become clear below, other social goals would indicate a different choice of technical methods for producing biological feedstocks, as well as refining and distribution procedures.

But trajectories are as much about communicating a vision as they are about realizing a set of material practices. A trajectory may also incorporate climate ethics *argumentatively* by including claims that rationalize, recommend or legitimize a given set of tools and techniques in ways that appeal to duties, responsibilities and cost/benefit calculations that are themselves defined and defended as a component of ethical imperatives that arise in connection with climate change. Other social goals would indicate a different set of ethical arguments, though of course distinct social goals can be related either as complements or as competitors. Advocates and opponents of biofuel alike pursued technical *and* argumentative strategies that were strongly influenced by the way that the future trajectory of biofuel was being envisioned and represented in journal articles, news reports and public relations campaigns. In particular, the mid-range time horizons (e.g., 10–25 years in the future) of carbon-mitigating trajectories were initially envisioned as logically compatible with short-term time horizons (e.g., 1–5 years) of trajectories with little or no commitment to environmental values. I argue that the merging of these ethically distinct trajectories in the public mind undercut support for biofuel R&D among environmentalists and that future attempts to pursue biofuels as a greenhouse gas mitigation strategy would do well to more forcefully articulate the ethical constraints on permissible development of biofuels more explicitly.

## Biofuels and climate ethics

The term “biofuel” is a relatively recent neologism. Usage in earlier decades covered a wide variety of organismal energy sources, such as “the biofuel cell” that was intended to power devices implanted in the body. It is today more generally used to indicate liquid fuels derived from plant-based sources that have been developed primarily for transportation utilization. Plant-based liquid transportation fuels have been identified as a means to mitigate harmful climate-related effects of fossil fuels. The emission-remediation argument for biofuel is both elegant and relatively simple. When petroleum-based fuels are burned, they release carbon and other greenhouse gasses into the atmosphere. The crude oil that is the source of most gasoline, diesel and kerosene liquid transportation fuels is mined from geologic sources, as is most natural gas. The carbon dioxide introduced into the atmosphere after the combustion of these fuels is now widely accepted to be altering the overall composition of atmospheric gasses, precipitating the processes known colloquially as climate change. When liquid transportation fuels derived from plants are burned, they also release carbon into the atmosphere. However, the process of plant growth removes carbon dioxide from the atmosphere through photosynthesis. Thus, the carbon released from burning of plant-based fuel is, in fact, being cycled through the atmosphere. It does not add to the proportion of carbon dioxide in the atmospheric composition and as such does not contribute to the processes that are thought to cause climate change.

Shifting a proportion of liquid fuel usage to sources derived from plants, including algae, has been identified as one of the climate stabilization wedges by the Climate Mitigation Initiative (CMI). A wedge is a technologically feasible strategy that will reduce one billion tons of emissions by 2060 (Pacala and Socolow 2004). CMI has identified eight such strategies and argues that if all were adopted aggressively over the coming decades, greenhouse gas emissions would be stabilized at current levels. The CMI biofuel wedge describes a 12-fold increase in ethanol production utilizing one-sixteenth of the world’s cropland (Princeton University, NJ, http://cmi.princeton.edu/). Because CMI limits itself to the existing technology, it does not examine the potential for new plant-based liquid fuels to make further contributions to a biofuels wedge. It thus may underestimate the potential contribution of biofuel in mitigation strategies. Nevertheless, the wedge concept is still useful because it illustrates how biofuels would be just one of the many mitigation strategies that would need to be pursued simultaneously. It is important to emphasize that even very ambitious targets for biofuel development envision the diversion of at most 15–20 % liquid transportation fuels from petroleum and natural gas to plant-based sources. Contrary to hyperbole that advocates biofuel as a “solution” to climate change (DOE, United States Department of Energy 1999), the development of liquid transportation fuels from plant-based sources is in no sense a panacea. However, the allure of a “carbon neutral” source of transportation fuels combined with the practical need for combining numerous partial solutions presents a compelling prima facie mitigation argument for the development of biofuels.

This prima facie mitigation argument provides a rationale for reviewing biofuel development strategies under the general heading of climate ethics. Since 2000, the enthusiasm for biofuels has waxed and waned. On the one hand, significant funds have been dedicated to research that would expand technological capability for the production and utilization of biofuels since 2000. This growth in research activity has been observed in both public sector institutions and in the private sector (Rajagopal et al. 2009). At the same time, the capacity to produce ethanol from the existing crops expanded considerably in the same period, especially in the largest producers of ethanol for transportation fuels, the United States and Brazil (Renewable Fuels Association 2011). On the other hand, biofuels and especially ethanol have suffered from negative press coverage since 2007 (Lomborg 2011; Rosenthal 2008). Opinion polls conducted since 2009 suggest that public support for policies to promote biofuel is soft and fragile (Anonymous 2009; Belden Russonello and Stewart 2011). Biofuel strategies for greenhouse gas mitigation seem to be thriving on the technical end, but may be flagging in terms of public support, providing a further rationale for considering the agricultural ethics of biofuels from a climate ethics perspective.

It is also possible that there will be climate-adaptation rationales that apply to biofuel. Here, the idea would be that development and utilization of biofuels would be undertaken in order to help counter some of the adverse impact of climate change. Within the context of agriculture, adaptation generally involves the development of new crops and new farming systems that will help farmers cope with changes in temperature, humidity and rainfall that are associated with climate change. In many cases, the challenge of adapting agricultural production will be extreme and may preclude all currently viable systems for agricultural production. In less drastic cases, farmers will search for crops (possibly including tree crops) that do well under changed climatic conditions. It is possible that crops useful for fuel production will be among them (Howden et al. 2007). However, the role that biofuels will play in adaptation is very unclear. The suggestion that growing crops for fuel rather than food will help farmers cope with climate change implies that the fuel crop will have unambiguous agronomic advantages over all food crops, but the current evidence for such a case is speculative (Smith 2010). What is more likely is that in a world with well-developed markets for biofuel, production of crops for fuel will prove more economically attractive than production of food crops for some farmers who have been harmed by climate change. However, to characterize such a scenario as providing an adaptation-oriented rationale for biofuels is to make an exceedingly broad claim. By this standard, virtually any economic development activity becomes an adaptation strategy. Until a more clearly articulated adaptation rationale is articulated, it is reasonable to presume that biofuels fit into climate ethics primarily as a mitigation strategy, though one should recognize that there may be other non-climate-related rationales that are ethically persuasive.

## The ethics of biofuels and the Nuffield Council report

The Nuffield Council on Bioethics is an independent organization based in the UK and established by the Trustees of the Nuffield Foundation in 1991. The Council is currently funded jointly by the Foundation, the Wellcome Trust and the Medical Research Council of the UK. The Nuffield Council has issued advisory reports on many issues in bioethics since its formation, including genetically engineered agricultural crops. For the report on biofuels, the Council’s approach was to summarize and categorize a number of distinct strategies for implementing plant-based liquid fuel production and to evaluate several detailed case studies in light of five guiding principles for biofuel development. The Nuffield guiding principles are as follows:

1. *Biofuels development should not be at the expense of people’s essential rights.*
2. *Biofuels should be environmentally sustainable.*
3. *Biofuels should contribute to net reduction of total GHG emissions and not exacerbate global climate change.*
4. *Biofuels should recognize the rights of people to just reward.*
5. *Costs and benefits of biofuels should be distributed in an equitable way* (Buyx and Tait 2011).

The Nuffield Council does not provide explicit criteria for prioritizing these principles, but the structure and pattern of argumentation in the report suggests a weak lexical ordering. That is, Principles 2 through 5 become relevant only when essential rights are protected, principles 3 through 5 are considered subject to satisfaction of both 1 and 2, and so on. The report focused on the existing strategies for producing ethanol and biodiesel from plant-based sources. Three such strategies have been extensively developed: ethanol from maize and sugarcane, and biodiesel from vegetable oils, including recycling of waste oils used initially in cooking or other industrial purposes. The Nuffield report also examines the emerging attempts to produce ethanol from crops such as miscanthus and jatropha, while a UNESCO report with similar findings studied palm oil production in Sarawak, Malaysia (Boonlong et al. 2011).

As indicated in the main report, current production of plant-based transportation fuels is the result of a complex blend of public and private initiatives. Many developed country governments have issued incentive programs that subsidize some of the cost of biofuel production, and some have established industry mandates for utilization of plant-based fuels that would be enforced by penalties if targets are not met. The Government of Brazil has been deeply involved in the development of ethanol production from sugarcane, issuing aggressive mandates for ethanol use, pioneering programs with publicly owned vehicles, negotiating favorable terms of trade for its biofuel industry, supporting the industry through EMBRAPA, the state-owned agricultural technology company, and maintaining land-use policies that are favorable to the production of sugarcane. In both the United States and Brazil, companies that refine plant feedstocks into ethanol are privately owned, and the ethanol supply is integrated into the existing privately run infrastructure for transportation fuels, and a similar structure exists for biodiesel production in Europe. Plant feedstocks are grown by independent farmers, though in almost all cases farm production units are comparatively large and in a few cases are corporately owned and managed. It is this ownership structure that accounts for a large percentage of the financial rewards from biofuels production accruing to relatively well-off individuals and groups (Nuffield Council on Bioethics 2011).

In light of the Nuffield report’s findings, the ethical problems associated with this pattern of public and private participation in biofuel R&D are symptomatic of capitalism and laissez-faire government policy. First, while carbon emissions mitigation joins classic job creation and economic growth rationales for the use of public funds to promote biofuels, implementation is embroiled in detailed matters that presuppose significant technical or policy expertise. In the 1930s, Walter Lippmann and John Dewey debated how the advocates of democracy should regard such issues, with Lippmann holding that the public will never be able to participate meaningfully in such matters while Dewey contended that true democracy demands that even technically complex decisions be open to a wide range of participatory input (Whipple 2005). For those who take Dewey’s position, biofuel decision making is not adequately democratic. Second, since the public contribution winds up channeling benefit disproportionately to private investors and to already large and powerful economic entities, it is questionable as to whether such policies are consistent with the spirit of John Rawls’ difference principle that holds that unequal distributions of benefit are justifiable only to the extent that provide the greatest possible advantage to the worst-off group (Rawls 1972). Finally, such classically capitalist decision-making structures provide very weak incentives or constraints that would conserve natural resources or protect ecosystem services (O’Conner 1993).

Commensurate with the criticisms published in scientific journals, the Nuffield Council report warns that some ethanol production may not, in fact, contribute to the reduction of greenhouse gas emissions associated with the use of petroleum-based fuels. The US ethanol industry is singled out for particular criticism on this score, as the fossil fuel energy consumed in the form of fertilizers, agricultural chemicals and fossil fuels burned by farm equipment and transport very nearly offsets all gains from the replacement of gasoline by ethanol for automobiles. When additional land-use effects are included in the analysis, the conversion of uncultivated lands to production for fuel makes ethanol from maize a net loser from the standpoint of mitigation (Nuffield Council on Bioethics 2011; Campbell et al. 2008; Righelato and Spracklen 2007; Sedjo 2008). Sugarcane production in Brazil is also questioned on the basis of land conversion, water use and environmental impacts associated with industrial methods of agricultural production (Nuffield Council on Bioethics 2011; Kennedy 2007).

The Nuffield report points out that rapid scale-up to industrial production standards can adversely affect small-scale producers. The Council notes that a Roundtable on Sustainable Biofuels (RSB) has been formed to develop and implement voluntary standards for environmental and social impacts from biofuel production. The RSB includes representation from smallholders and has received recognition from umbrella certification groups that have previously undertaken environmental and social justice initiatives. The Nuffield report also takes notice of concerns about the food versus fuel tension. As mentioned above, biofuels suffered a major loss in prestige when major international press organizations began to attribute rising hunger to the diversion of agricultural commodities for ethanol production in 2008 (Thompson 2009). The Nuffield report holds out hope that the avoidance of food crops such as maize can be an effective means to manage the food versus fuel conflict.

Succinctly, the Nuffield Council found current production of biofuels to be lacking on virtually all of its five criteria, but nevertheless found the overarching mitigation rationale summarized above to be compelling. As such, the authors of the Nuffield Council report recommend the establishment of a European council that will monitor biofuel programs to measure compliance with the five principles (Tait 2011). The report praises voluntary efforts such as the RSB and encourages private sector firms to adopt and follow these guidelines. However, the strategies reviewed by the Nuffield Council are sometimes referred to as “first-generation biofuels.” In contrast, there has been a significant research investment in new technologies that would contribute to the biofuels wedge in climate change remediation strategies. Reviewing the climate ethics of biofuels thus requires a more concerted effort to envision the future scenarios of biofuel development. It is in this connection that the conception of a technological trajectory may prove helpful.

## A passel of trajectories

According to Don Ihde, a trajectory is an imaginative scenario that envisions a particular course of development for a technology and that is especially relevant to the way that actors of various sorts will align into networks either in support of the trajectory or in opposition. For Ihde (1990), the trajectory metaphor fixes our attention on the way that technological innovations will unfold over an indefinite future in which many unknown and unknowable events will affect their design, implementation and utilization. Both new and old approaches to biofuel development are usefully analyzed in terms of the trajectories that various actors envision for their implementation (Thompson 2008). Although it is not entirely clear where the terminology of technological trajectories originates, an early usage comes from the discipline of economics. Nelson and Winter (1982) defined a technological trajectory as the natural course of evolution in technical change. Their work was a contribution to a then-recent interest among economists in understanding how technological innovations might be understood as “endogenous,” or arising in response to scarcities reflected in the operating environment of firms producing within a given economy. In contrast, earlier work had presumed that important technological innovations bore little relationship to the price of inputs or the operating costs of firms (see Pearce and Barbier 2000, p. 32 for a discussion of the environmental significance of this debate).

Of course, the use of such terminology in a definition invites critique. Frank Geels takes issue with Nelson and Winter’s use of the word “natural” citing Donald MacKenzie to the effect that a technological trajectory is merely a self-fulfilling prophecy. According to Geels (2007), “technologies develop along trajectories because engineers share cognitive rules (ideas, perceptions, beliefs, expectations) that guide their activities in certain directions. Trajectories are not natural but performed. Hughes would add that trajectories are not only stabilized by beliefs but also by social and technical linkages, vested interests, regulations, infrastructures, and so on.” Although my use of the expression is not incompatible with Geels’ observations, it is derived from Don Ihde who uses it to emphasize the way that a technology developer’s initial vision and intention are subsequently affected by forces in the social and natural environment, just a projectile’s actual trajectory is affected by wind, rain or a deflecting object. As Geels notes, trajectories reflect ideas, perceptions, beliefs and expectations, but the term emphasizes the way that all of these interact with other forces (including other actors) in the socio-technical environment. A trajectory may thus fall short of fulfilling the expectations indicated by engineers’ shared cognitive rules.

As noted in my 2008 paper, climate mitigation strategies are far from being the only or even the most persuasive trajectories that influence the formation of networks around biofuel development. In addition to the longstanding (and very slow) push for chemurgy that dates back to the days of Henry Ford and the lingering romance of appropriate technology, biofuels were envisioned and advocated by a number of groups between 2000 and 2010. Although each of these groups saw biofuel being developed and its usage growing over the coming years, they differed markedly in the underlying reason for pursuing biofuel technologies, and as a result, often envisioned somewhat distinct futures for biofuel development. Bruno Latour’s (2005) actor-network theory can be utilized to understand how opposition and promotion of biofuels has had as much to do with the way that networks formed around several key trajectories. The following discussion omits empirical sociological analysis and relies on an intuitive understanding of how envisioned futures might align actors into networks and counter-networks around particular configurations of biofuel possibilities.

### The triple-bottom line

The mitigation argument can be seen as the latest and perhaps most important point in a trajectory that has long-envisioned biofuels as making an important contribution to environmental objectives. Biomass in the form of firewood has been used as an energy source since antiquity, and prior to the development of steam and internal combustion engines, much of the energy dedicated to transportation was derived from plant-based sources in the form of animal feeds. What is more, the idea of refining plant biomass for liquid transportation fuels has been actively pursued since the earliest days of the automobile (Finlay 1990, 2003). Plant-based sources of energy were discussed with renewed enthusiasm in the wake of an “appropriate technology” movement that swept the globe in the 1960s and 1970s (Lovins 1977). The use of methane generators powered by biological waste materials was imagined as an inexpensive and environmentally sensitive alternative to centralized systems for refining fuels or generating electricity (Brown 1978). Well into the decade of the 1980s, bio-based energy sources were viewed as attractive ways to mitigate environmentally damaging impacts associated with fossil fuels (Williams 1985).

As the previous discussion of the Nuffield Council report makes clear, the turn to mitigation is strongly coupled with a conception of sustainability that is reconciled to the idea that for-profit entities must more than recover their costs in order to be economically sustainable. And public sector entities cannot undercut the private sector without destroying the employment and tax base that is crucial to the economic sustainability of society as a whole. As such, pursuit of environmental sustainability—here expressed in terms of mitigation—must go hand in hand with economic sustainability. The idea of the triple-bottom line is to harness efforts at social betterment, human rights and the pursuit of justice to the economy and the environment in the search for economic and technological endeavors that are positive in all three dimensions (Rogers and Ryan 2001). Thus, the aim of the Nuffield Council and of the RSB can be accurately characterized as harnessing biofuels development to a triple-bottom line conception of sustainability in which private sector innovation is undertaken in partnership with governance activities undertaken by voluntary non-profit organizations, as well as state-based regulatory agencies.

### Energy independence

My earlier paper notes the strong support for biofuel development being advocated by then US President George W. Bush and his Secretary of Agriculture Mike Johans. Speaking at a World Agricultural Forum meeting in 2007, Johans was uncompromising in committing the Bush administration to all manner of policies that would promote the development, uptake and utilization of biofuels. Although Johan’s background as the former Governor of Nebraska (a corn state) might have aligned with those advocating alternative uses for maize on economic grounds (discussed below), the political rhetoric behind Bush administration support for biofuel emphasized ending US dependence on imports of foreign oil. The unsubtle subtext of this rhetoric implied a linkage between energy policy and the administration’s “war on terror.” It exploited the resentment of US reliance on petroleum products sourced from the Arab world and implicitly enlisted patriotic feeling still resurgent in the wake of September 11, 2011 attacks on the World Trade Center in New York City (Thompson 2008).

Energy independence became a political theme with significant popular appeal in the United States as a way to isolate the US economy from supply distortions believed to be caused by the Organization of Petroleum Exporting Countries (OPEC). It combines an economic rationale with patriotic concern over the putative power wielded by OPEC nations. The trajectory includes the expansion of domestic petroleum production as well as novel methods for the extraction of natural gas (such as fracking) and is thus fairly unspecific in the particular form or type of fuels that would be developed. As ethanol came to be included as an auxiliary to domestic oil exploration, the advocates of energy independence have been vague about the biomass feedstocks that would be converted to ethanol. Bush suggested that switchgrass would provide a long-term source of biomass for the biofuels in his 2006 State of the Union Address (Lewin 2006). In the United States, both political parties eventually embraced the political framing of energy independence. The bill that created a tangle of economic incentives for corn ethanol production was originally introduced as the Clean Energy Act but was renamed and passed as the Energy Independence and Security Act of 2007 (P.L. 110–140) under the leadership of Democrat Nancy Pelosi. The theme of energy independence is also discussed for Europe, though in light of Europe’s reliance on Russian oil, the discussion lacks some of the “war on terror” overtones that Bush was able to exploit in the United States (Artens 2008; Brower 2010).

### Corn ethanol forever (or the Hopi ritual)

Some time back, I heard the well-known sociologist Cornelia B. Flora introduce a talk by describing religious practices among the Hopi and the Tzotzil civilizations, both of whom describe themselves as “the people of the corn.” Flora described how numerous dances, prayers and rites were performed to alert the tribal farmers that spring was in the air and to insure that they did not fail to plant their corn. “But the Hopi had nothing on us in Iowa,” continued Flora, who went on at some length to detail a long list of Federal subsidies, crop insurance, State tax incentives and other assistance programs, all seemingly designed to insure that Iowa farmers did not forget to plant the corn. To my knowledge, Flora has (perhaps wisely) never published this little vignette, but her point is a highly appropriate lead into what has perhaps been the dominant trajectory for biofuel in the United States for many years.

Producers of any commodity have a basic economic incentive to find alternative uses for said commodity, as alternative uses provide an additional source of economic demand. The incentive is especially strong for agricultural producers, as food commodities are notorious for the inelasticity in their demand curves. That is, whatever the supply, people need to eat roughly the same amount of food. This means that consumption (hence demand) does not rise as people become wealthier, as it might for electronic appliances, entertainment or even education. Corn growers have been able to enjoy some elasticity in the demand for their product because it is used as an animal feed, and as wealth increases, people do increase their consumption of animal protein. In recent years, corn has also been converted into other food ingredients, most notably high fructose corn sweeteners, which are used widely in sweetened beverages. Nonetheless, corn growers in the United States have been especially assiduous in promoting alternative uses for their crop. In this connection, the Corn Ethanol Forever trajectory becomes linked to the Energy Independence trajectory through the passage of the aforementioned Energy Independence and Security Act of 2007. The convoluted subsidies paid under this law provide even more reason to rest secure in the faith that US farmers will not forget to plant the corn. An analogous trajectory might be traced for ethanol derived from sugarcane.

### Out of thin air

The trajectory that generates the most enthusiasm in scientific circles is actually a complex tangle (Andrew Pickering would say “mangle” Pickering 1995) of discrete research programs dedicated to technology R&D. Chief among these programs are attempts to provide a practical method for producing cellulosic ethanol. All plant matter contains the sugars that are the basis for ethanol production, but in many plants, these sugars are bound within the sturdy cell wall. The energy and processing currently needed to break down the cell wall and access these sugars makes it economically infeasible to produce ethanol from all but a few plants such as maize and sugarcane. However, if technology can overcome this problem, biomass feedstocks for ethanol production could be derived from virtually any plant, including fast-growing trees that might be produced on lands unsuitable for crops. Advocates of cellulosic ethanol argue that this would substantially reduce any ethical concerns about a conflict between food and fuel usage (Lynd et al. 2008). The technical strategies for unleashing cellulosic ethanol include genetic engineering of plants to make their cell walls more amenable to processing, genetic engineering of microbes that have greater efficiencies in breaking down plant cell walls and other strategies utilizing biochemistry and synthetic biology (Sticklen 2008). Although cellulosic ethanol is the holy grail of second-generation biofuels, other strategies are also being pursued. One is the use of advanced plant breeding and biotechnology to increase the oil content in crops used to produce biodiesel (Dyer et al. 2008). Another would build on the rapid growth and efficiencies of algae, though the current understanding of the complex genomes in algae limits the short-term promise of this approach (Hu et al. 2008). The construction of wholly synthetic genomes is also being pursued in connection with biofuels (Galperin 2008).

These second-generation biofuels promise greater efficiencies and may alleviate some of the environmental impacts that have been associated with ethanol production from maize and sugarcane feedstocks. They are, moreover, classic instances of innovation-led trajectories. Successful movement from the laboratory to a practical biofuel will require the alignment of a complex network that includes scientists, financial leadership, government funding agencies, venture capitalists and engineers who will be needed to tackle the scale-up and production issues that will loom large as promising technologies near the prospects of commercial release. In this respect (if not also the use of genetic engineering), this trajectory is also a classic instance of agricultural biotechnology. As such, securing the protection of intellectual property will certainly be a crucial component in the Out of Thin Air trajectory. At the same time, public uncertainties about the environmental release of transgenic organisms and established constituencies opposing the biotechnology industry insures that the Out of Thin Air network will be opposed by a somewhat organized and highly motivated counter-network, already prepared to thwart them.

## Trajectories and climate ethics

The four trajectories described above are philosophical constructs, though they have not been put together in the absence of supporting data. One might question whether they accurately describe the landscape of biofuels development as the world moves deeply into the second decade of the third millennium. A useful discussion of the empirical validity of these constructs would require sociological results that are not currently available. In the philosophical discussion that follows, they will be taken at face value and will be assessed in terms of ethical commitments and possible pitfalls. As such, the emphasis here is on the way that implicit trajectories play a role in structuring networks of actors. My discussion emphasizes the way that human beings, organizations and technological artifacts moving along each one of the four trajectories are quite likely to collide with actors and actants moving along a different one. At the same time, my primary interest lies in the way that actors take themselves to be justified by ethical principles, including (but not limited to) the principles enunciated in the Nuffield Council report on biofuels.

In fact, only Triple-Bottom Line has well-characterized ethical principles at all, at least as they bear on biofuels. Characterized aptly by the principles articulated in the Nuffield Council report, this trajectory hopes to blend environmental goals such as mitigation and sustainability with distributive justice, human rights and fair play. These classically liberal political values undoubtedly enjoy widespread appeal, but they are historically countered by those who doubt they can be realized without placing the economic and political survival of a society at risk. In just that vein, Energy Independence maintains an implicit commitment to a nationalistic conception of self-reliance and is occasionally tempted by explicit appeals to patriotism and defense of national identity. As noted already, these values apply to biofuels only to the extent that a biofuels policy happens to coincide with the development of domestic sources for the production of liquid fuels. But since the boundaries of a nation coincide with the lands on which agricultural production occurs, there may be a permanent concordance between the ethical commitments of Energy Independence and the interests of agricultural producers. This suggests a natural alliance with Corn Ethanol Forever. On its own, Corn Ethanol Forever has little going for it in ethical terms beyond the fact that one violates no existing laws in growing corn or in lobbying for policies that serve one’s economic self-interest. The connection of farm and national interests is a mainstay for Jeffersonian agrarianism (Thompson 2010). Finally, to the extent that Out of Thin Air is committed simply to technology development, its core value would appear to be a form of faith in the inherently progressive nature of scientific achievement. Out of Thin Air justifies itself as a manifestation of the Enlightenment Ideal: the belief that growth in knowledge cannot but help benefit mankind as a whole, and that when harnessed to technological innovation, capitalism’s voracious pursuit of profit is turned to social benefit.

In order to explore the vulnerabilities created by this commitment to the Enlightenment Ideal, it may be useful to begin with a collision that has already occurred. Back in 2007, Energy Independence and Corn Ethanol were enjoying enormous political success in the United States, seen notably in the Republican Bush administration’s vocal support and in the passage of the Energy Independence and Security Act in a Congress controlled by the Democratic Party. The US technology sector enjoyed some modest benefits, but there is nothing in these two trajectories that links them tightly to Out of Thin Air. Indeed, to the extent that any of the new technologies being pursued in that trajectory are realized, they become competitors to corn ethanol, creating a tension with the corn growers who constitute the spine of Corn Ethanol Forever. However, this tension was neatly mitigated by a blended trajectory concocted from the elements of all three. The subsidies and massive expansion of ethanol production facilities could be rationalized as temporary measures that serve the immediate goals of Energy Independence, while also developing infrastructure for Out of Thin Air. At the same time, an alliance with Out of Thin Air provided corn growers and Energy Independence types with a way to forestall environmental critics who were already skeptical of the energy balance obtainable from maize-based ethanol. Corn growers were willing to accept policies that project events adverse to their profits as long as those events are several years in the future. Their experience with a long series of US farm bills had taught them to grab a government paycheck while you can get it, because it may well be possible to reverse the adverse policy when the future actually arrives (Bonnen et al. 1996).

The idea that second-generation biofuels needed infrastructure was a key element in this blended trajectory. It was recognized that the ability to utilize ethanol from *any* source was constrained by the lack of filling stations that could deliver higher blends of ethanol fuels (such as E 85, a blend of 85% ethanol and 15% gasoline). What is more, the American fleet of vehicles needed a larger percentage with engines that could burn such fuels if the targets envisioned in the Energy Independence and Security Act were to be met. Given the recognition that it would take years for this infrastructure to be developed, advocates of second-generation biofuels were able to rationalize the costly and environmentally unsustainable incentives and mandates that were included in the Act (Tilman et al. 2009). In doing so, actors aligned by Corn Ethanol Forever and Energy Independence were able to enlist the prestige and expertise of the scientific community in their cause.

However, it is questionable as to whether this alliance was either politically wise or ethically justified for Out of Thin Air. Expansion of corn production on the Great Plains has grown in lockstep with the construction of new ethanol production facilities in the Dakotas, Western Minnesota and Nebraska. In many of these areas, corn is being grown in 2009–2011 on grasslands that have not only been carbon sinks, but have been prime habitat for native plant and animal species (Johnson and Stephens 2011). In the meantime, scientists who were enlisted in the mixed trajectory compromise have advocated biofuels harvested from native prairie grasses, rather than maize, in the same areas where corn production has expanded (Fargione et al. 2008). Although it is virtually impossible to measure the public’s enthusiasm for obscure scientific research projects, much less to gauge quantities as amorphous as the prestige of science, it seems reasonable to suspect that advocates of Out of Thin Air will have a much more difficult time recruiting ordinary people into their network than they did in 2007.

It is also possible that scientists in the Out of Thin Air network have undercut some of the conservation-oriented ethical values to which they themselves were committed. First, as the Nuffield report indicates clearly, in reconciling themselves to policies that promote corn ethanol, they become implicit supporters of a technology with negative climate impact. Even if the Out of Thin Air scientists see this as a temporary and even useful evil, they have nonetheless allowed less environmentally laudable rationales for promoting biofuel to dominate the public discourse. Thus, second, they have arguably reduced the public’s capacity for understanding and evaluating the environmental rationale for biofuels. If so, then the third and most significant way in which their temporary alliance with Corn Ethanol Forever harms conservation goals is that they may have actually *increased* the barriers to the adoption of biofuels that are truly beneficial to mitigation strategies.

Finally, if it is correct to say that Out of Thin Air has relied entirely on the Enlightenment ideal of progress, then the R&D component becomes vulnerable to the main thrust of philosophical and political criticism that has been leveled against science and technology for over 100 years. The list of theorists who have made this critique begins with Karl Marx and includes Herbert Marcuse, Hans Jonas, Richard Sclove, Andrew Feenberg and Langdon Winner. While Marx and Marcuse may have presumed that socialism would be the primary corrective to this tendency, others on this list have been deeply attentive to the way that when nominally socialist governments in the USSR and China became dominated by engineers, they tended to make many of the same mistakes. Thus, a primary thrust of recent work in the philosophy of technology has stressed the need for R&D to be informed by a critical and ethically oriented inquiry that probes the possible configurations of a technical system and that seeks means to implement the technical systems that empower rather than weaken individuals and social groups that are currently disadvantaged and that give voice to a wide range of potentially affected parties at various stages throughout the process of research and development (Feenberg 1991; Jonas 1984; Sclove 1995; Winner 1986). Although references to these philosophers are absent, the Nuffield Council’s recent report is best read as a continuation of that tradition.

## Concluding ethical findings and speculations

The “technological trajectory” construct relies on the metaphor of physical objects moving through time and space as a result of momentum imparted initially or continuously combined with resistance and deflections that arise in connection with the environment in which they are moving (including other projectiles). Although the metaphor has been criticized in science and technology studies, the metaphor of momentum aptly conveys the sense of propulsion and force that has tempted analysts and ordinary people alike to use the language of technological determinism. And it does so while acknowledging the possibility of intervening forces and events. At the same time, the notion of a trajectory implies an “aim” or “direction” that calls to mind the way that technologies are developed and deployed by people’s objectives and intentions for doing so. This aspect of a trajectory is well suited to ethical analysis, for it puts normativity—the potential for evaluation in terms of correctness, morality, justice and acceptability—into the foreground. Yet, the idea of a trajectory is also convenient in that it permits the ethical analyst to discuss this normativity as a feature of the vague and general way that technologies are heading (and the place they are envisioned to end up) without also having to impute specific ethical intentions, aims or values to the human beings who are involved in their development and implementation. This is not to say that such imputations are never justified, but there are also occasions where it is important to discuss and examine ethical rationales and outcomes from the development or implementation of tools and techniques in abstraction from the specific activities or motives of the human beings who are the agents of development and implementation.

In the case of emerging technology for biofuels, technology that may well deploy applications of biotechnology, nanotechnology and synthetic biology, the eventual tools and techniques that emerge will be entering a commercial and political space in which plant-based sources for liquid fuels have been operating for some time. The atmosphere in which the trajectory for emerging biofuels must move continues to be highly contested, and the underlying assumptions of models and projections drive much of the debate. My original 2008 paper argued that although epistemological issues and questions of logical consistency surround and interpenetrate the debates over models and projections, philosophical engagement with these epistemic questions is unlikely to have much influence over the course of the debate. The actor-network politics of technological trajectories largely explains why this is the case. It is either the projected end-point of a trajectory that is envisaged by advocates and detractors, or the way that the actors are displaced through the unfolding of a trajectory that enrolls actors into both networks and counter-networks. Mounting arguments about whether a given endpoint or displacement will actually be realized becomes part of the political give-and-take between network and counter-network, but close scrutiny of the epistemological assumptions underlying these arguments would have to lend one side or the other a decisive advantage in order to have a significant impact on network politics. The enduring nature of philosophical questions makes this unlikely, and the “excess of objectivity” noted by Dan Sarewitz (2004 and discussed in Thompson 2008) ensures that there will always be an opportunity for another study to purportedly “resolve” these seemingly empirical and technical questions.

For example, a recent critique of biofuels regulations discusses both US and European regulatory standards for renewable fuels, arguing that the United States unjustifiably neglects quantifiable inefficiencies in biofuel reduction of greenhouse gas emissions (in comparison to gasoline) that accrue as a result of differences in farming practice and geographical locale (Fast et al. 2011). This critique builds upon earlier criticisms of ethanol that called attention to the negative impact of clearing forests and native grasslands for corn or sugarcane production (Fargione et al. 2008). Yet, the new critique also argues that life cycle analysis, the key analytic tool that has been used for assessing biofuel impacts on climate change, is particularly ill-suited to the assessment of environmental impact from agriculture owing to the high degree of variability in production practices and their local environmental costs (Fast et al. 2011). The paper fails to deliver a knockout blow to the Corn Ethanol Forever network precisely *because* it opens a space for a new round of analyses and methodological refinements in one of the primary analytic tools—life cycle analysis—being used to make projections. As my 2008 paper predicted, there is literally no end to refinements and epistemic critiques that can be leveled against these environmental assessments. The emerging technologies must thus chart a trajectory through a social discursive space that will be thick with a highly detailed and technically complex residue of argumentation. At the same time, the very density and complexity of that argumentation eventually undermines its own effectiveness. When that happens, it is the relative political and economic power of actors enrolled in the network that really matters.

But as the give-and-take between actors aligned on colliding trajectories moves more clearly into the political sphere, the ethical rationales supporting a trajectory have the potential to become important. As political scientist Kristen Magis has argued, despite little or no standing in state-based or intergovernmental decision making, the advocates of global civil society have been able to win important concessions from both governments and for-profit firms by mounting their arguments in terms of social justice, equity and sustainability (Magis 2009). The ability to align a trajectory with some ethically persuasive rationale may thus prove to be crucial to the actors’ ability to prevail in contests with actors from counter-networks. And as ethical rationales come to the fore, the way in which actors aligned by different trajectories come to see themselves as having strategic goals in common can change.

Gazing into the crystal ball, it would appear that in the future Out of Thin Air will be more comfortable being aligned with Triple-Bottom Line than with any other extant trajectory. The compatibility of these trajectories rests on ethical more than technical grounds. Both of these trajectories place the mitigation argument for biofuels front and center in conceptualizing future scenarios, while mitigation is not a central component of the reason for developing biofuels for either Energy Independence or Corn Ethanol Forever. However, in order to achieve a melding of Out of Thin Air with Triple-Bottom Line, researchers in both public and private sectors may need to embrace the Nuffield Council’s call for monitoring bodies. And scientists have never been comfortable with non-scientists looking over their shoulder. Prior scuffles between plant scientists and the public over agricultural biotechnology would quite reasonably make scientists engaged in second-generation biofuels work even more reluctant to accept a governance process in which environmentally oriented non-scientists were projected to play a crucial role.

At the same time, this juxtaposition of trajectories also provides some basis for those who support the Nuffield Council recommendations to step back and reconsider the envisioned trajectory of Triple-Bottom Line. The Nuffield recommendations call for standards, monitoring and certification by third parties as a mode of ethics governance. The model has enjoyed at least limited success in promulgating products under labels such as “organic” and “fair trade,” and some research supports its ability to secure improvements in the quality of life or participation in governance processes (Bacon 2010; Valkila and Nygren 2010). However, the same research also reveals continuing vulnerability to exploitation among smallholders, and other researchers have been far more critical of the concessions that standards and certifications processes make to neoliberal state institutions and for-profit firms (Goodman 2010). One weakness of such processes is that “ethics” becomes a matter of compliance to standards, effectively vitiating the idea that ethics should be understood as an open-ended form of philosophical inquiry (Haggerty 2004).

An alternative might be fashioned in terms of an activity in which the actors enrolled in Out of Thin Air and Triple-Bottom Line might actively engage in ethical reflection and debate. Here, ethics would be conceptualized less as an activity of compliance and more as a form of self-governance through collaborative inquiry. The upshot of an ethical review of biofuels might indeed lead to more formal processes of standard setting and certification and perhaps might extend to governmental regulation and policy setting as well. Yet, these activities would not themselves be understood as the inevitable result of an ethical evaluation, much less part and parcel of what a “climate ethics of biofuels” would entail. More specific suggestions for engaging the science community in such ethical inquiry abound: They include highlighting the normative aspects of engineering design (Whitbeck 2011), adaptations of discourse ethics (Korthals 2004; Thompson 1999) and the “ethical matrix” developed originally for engaging scientists in ethical reflection by Ben Mepham (Cotton 2009; Mepham 2000). By utilizing one of these methods to engage ethical issues, actors in Triple-Bottom Line and Out of Thin Air might well discover deeper commonalities of vision (though they also might not). It is a way of proceeding in ethics that eschews dicta and pronouncement and seeks instead for probes that draw people in. It is in that spirit that a vague, provocative and perhaps even playful analysis of biofuels, trajectories might contribute to a happier convergence of future scenarios for emerging technology.
